# Fibroblast growth factors induce hepatic tumorigenesis post radiofrequency ablation

**DOI:** 10.1038/s41598-023-42819-2

**Published:** 2023-09-28

**Authors:** Aurelia Markezana, Mor Paldor, Haixing Liao, Muneeb Ahmed, Elina Zorde-Khvalevsky, Nir Rozenblum, Matthias Stechele, Lukas Salvermoser, Flinn Laville, Salome Goldmann, Nofar Rosenberg, Tomas Andrasina, Jens Ricke, Eithan Galun, Shraga Nahum Goldberg

**Affiliations:** 1 The Goldyne Savad Institute of Gene and Cell Therapy, Hadassah Hebrew University Hospital, Ein Karem, Jerusalem, Israel; 2 Laboratory for Minimally Invasive Tumor Therapies, Department of Radiology, Beth Israel Deaconess Medical Center (BIDMC), Harvard Medical School, Boston, MA USA; 3 Division of Image-Guided Therapy and Interventional Oncology, Department of Radiology, Hadassah Hebrew University Hospital, Jerusalem, Israel; 4 Department of Radiology and Nuclear Medicine, University Hospital Brno and Masaryk University Brno, Brno, Czech Republic; 5 Department of Radiology, University Hospital, LMU Munich, Munich, Germany

**Keywords:** Cancer therapy, Oncology

## Abstract

Image-guided radiofrequency ablation (RFA) is used to treat focal tumors in the liver and other organs. Despite potential advantages over surgery, hepatic RFA can promote local and distant tumor growth by activating pro-tumorigenic growth factor and cytokines. Thus, strategies to identify and suppress pro-oncogenic effects of RFA are urgently required to further improve the therapeutic effect. Here, the proliferative effect of plasma of Hepatocellular carcinoma or colorectal carcinoma patients 90 min post-RFA was tested on HCC cell lines, demonstrating significant cellular proliferation compared to baseline plasma. Multiplex ELISA screening demonstrated increased plasma pro-tumorigenic growth factors and cytokines including the FGF protein family which uniquely and selectively activated HepG2. Primary mouse and immortalized human hepatocytes were then subjected to moderate hyperthermia in-vitro, mimicking thermal stress induced during ablation in the peri-ablational normal tissue. Resultant culture medium induced proliferation of multiple cancer cell lines. Subsequent non-biased protein array revealed that these hepatocytes subjected to moderate hyperthermia also excrete a similar wide spectrum of growth factors. Recombinant FGF-2 activated multiple cell lines. FGFR inhibitor significantly reduced liver tumor load post-RFA in MDR2-KO inflammation-induced HCC mouse model. Thus, Liver RFA can induce tumorigenesis via the FGF signaling pathway, and its inhibition suppresses HCC development.

## Introduction

Image-guided thermal tumor ablation, including radiofrequency (RF) systems, are commonly used to treat focal primary and metastatic tumors in the liver, lung, kidney, bone, and other sites^1–3^. Advantages of thermal ablation, including minimal-invasiveness, low morbidity, and cost-effectiveness, have led to its rapid adoption and clinical implementation^1,4^. Despite advantages of RF ablation (RFA) over surgery, 5-year survival rates remain approximately 50%, with 70–81% new tumor recurrence in hepatocellular carcinoma (HCC)^3,5–7^. Indeed, there is ever increasing evidence that local hepatic RFA can promote tumor growth at a distance from the ablation site^8^. Thus, strategies to suppress local and systemic pro-oncogenic effects of RFA are urgently required to improve therapeutic effects and clinical outcomes.

Recently, we have shown that this presumably local therapy induces systemic effects including potentially unwanted “off- target” tumorigenesis even when ablating only normal tissue^8,9^. Specifically, ablation of normal liver can cause distant mammary tumor (R3230 and MATIII) to grow in rats, and increase HCC tumor burden in the MDR2-KO mouse^8,10^. Additionally, partial ablation of a tumor increases growth of distant tumor^11^. Dramatic increases in serum IL6 have been reported after RFA in animal and clinical studies^12,13^. Moreover, colorectal cancer (CRC) patients with elevated serum HGF and IL-6 post-RFA are associated with tumor progression, metastases, and poor survival^12^. Several cytokines, growth factors, and inflammatory mediators with potential pro-tumorigenic properties, including, HGF, STAT-3, and VEGF, increase following RFA^11,13–16^. These and additional factors are expressed either by parenchymal or recruited cells. Indeed, following liver RFA an increased inflammatory response has been observed including cellular recruitment of neutrophils, macrophages, and activated myofibroblasts to the ablated zone^14,17^. Nevertheless, the main mechanistic pathway that drives RFA's pro-oncogenic effects has yet to be fully elucidated.

It is critical to uncover the key pro-tumorigenic mediators and their cellular origins in order to develop optimal strategies to suppress the pro-oncogenic effects of RFA. For example, partially heat stressed parenchymal hepatocytes, similarly to residual tumor, may produce the cytokines implicated in the RF-induced tumorigenesis cascade^11^.

The purpose of this study is to determine mechanisms of post-RFA tumorigenesis induced by hepatocytes, by identifying potential cytokines and growth factors that they produce which may play key roles in the initiation of tumorigenesis post-RFA. To do this, we investigated human samples and reinforced our hypothesis using an in-vitro model of RFA and by performing in-vivo mouse studies modeling RFA using an FGFR inhibitor to confirm our results.

## Results

### Plasma from patients post hepatic RFA induce variable proliferation of HCC cell lines by activation of multiple cytokines and growth factors including FGF

**Figure 1:**
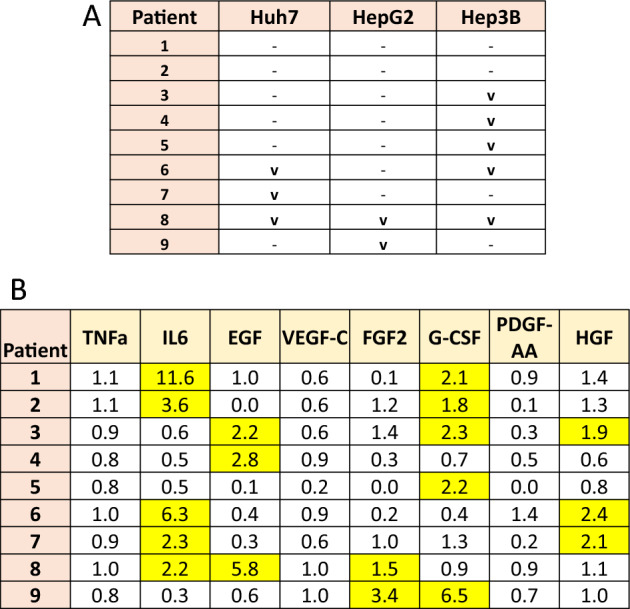
Plasma from patients post hepatic RFA variably induce proliferation of HCC cell lines associated with an increase in cytokines and growth factors. (**A**) To determine whether hepatic RFA induces secretion of pro-tumorigenic factors, human HCC cell lines were incubated with 20% plasma of nine HCC (n = 3/9) and CRC (n = 6/9) patient baseline and 90 min post-RFA plasma. Live continuous cellular proliferation (marked as V) was monitored using an IncuCyte device. (**B**) Plasma of patients induce statistically significant increased proliferation (marked in yellow) in some HCC cell lines 90 min post-RFA treatment (FC). Three samples induced proliferation of Huh7 with protein analysis showing elevated levels of IL-6 and EGF or HGF compared to pre-RFA. Five cases of proliferation of Hep3B were seen, most having increase of EGF or HGF without IL-6 increase compared to pre-RFA. Two patients induced proliferation of HepG2 had elevated levels of FGF2 compared to pre-RFA. All measurements were performed in triplicate.

Human HCC cell lines were incubated with 20% plasma of HCC (n = 3) or CRC (n = 6) patients before and after RFA treatment and subjected to cellular proliferation monitoring using the IncuCyte device. This plasma induced statistically significant increased proliferation in some, but not all of the tested HCC cell lines 90 min post-RFA treatment compared to baseline pre-RFA plasma. Specifically, three samples (33%) induced proliferation of Huh7, five (55%) caused proliferation of Hep3B, two (22%) induced proliferation of HepG2, and two samples didn’t induced proliferation of any tested HCC cell line (Fig. 1A).

Multiplex Elisa analysis (milliplex) of the samples showed elevated levels of IL-6 and EGF or HGF compared to pre-RFA in samples that induced proliferation of Huh7. Specifically, plasma of patient 6 and 7 had elevated levels of IL-6 and HGF (6.3, 2.4-FC and 2.3, 2.1-FC, respectively). In patient 8, high levels of IL-6 and EGF were detected (2.2 and 5.8-FC, respectively). Four samples that increased Hep3B growth had increased levels of EGF or HGF with three having no IL-6 increase compared to pre-RFA. Patient 3 had an increase in both EGF and HGF levels (2.2- and 1.9-FC, respectively). In patients 4 and 8, increased levels of EGF were detected (2.8- and 5.8-FC, respectively). The fifth sample that induced HepG3 had elevated G-CSF levels (2.2FC). Both of the samples inducing HepG2 proliferation had elevated levels of FGF2 compared to baseline (patients 8 and 9; 1.5- and 3.4-FC, respectively). Samples not inducing proliferation only had elevated levels IL-6 and G-CSF with no increase in other growth factors detected (Fig. 1B).

### FGF, IL6, and other growth factors detected in human post-ablation plasma induce proliferation of HCC cell lines in-vitro

**Figure 2:**
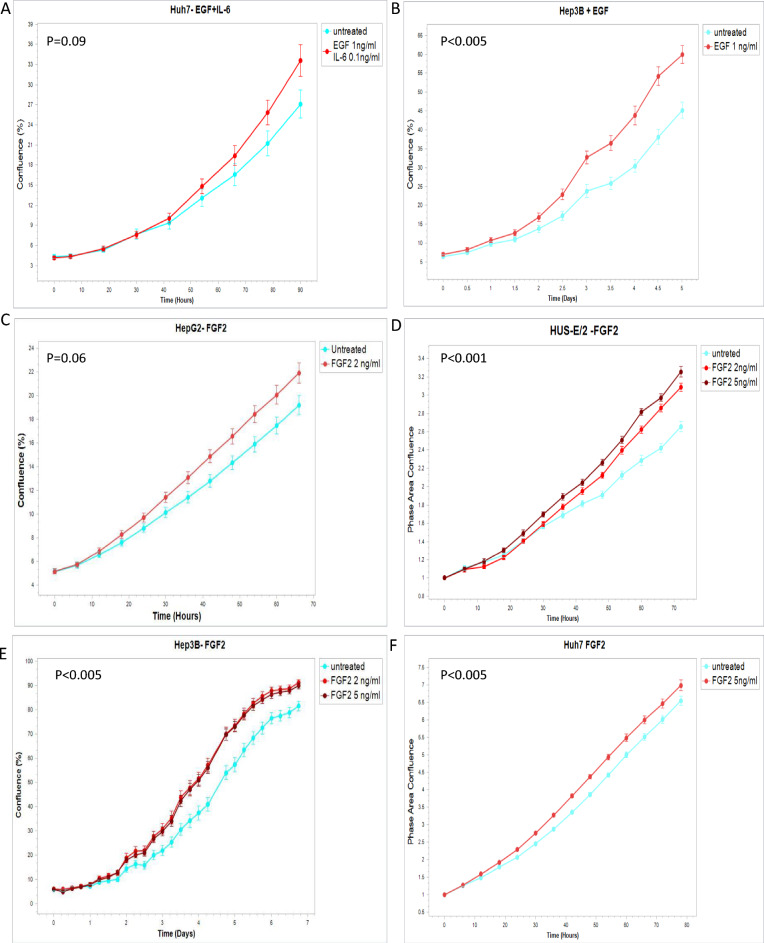
Selected proteins increased in post-ablation plasma induce proliferation of HCC cell lines In-vitro. Key proteins elevated in patient plasma were tested on three human HCC cell lines by incubating the cells with recombinant proteins. (**A**) Addition of 1 ng/ml EGF with 0.1 ng/ml IL-6 to the growing medium of the cells exclusively induced proliferation of the Huh7 cells. (**B**) EGF exclusively induced proliferation of Hep3B. (**C–E**) However, FGF2 induced proliferation in all the HCC cell lines tested. (**F**) Moreover, FGF2 induced proliferation of a fourth Hus-E/2 cell line (i.e., immortalized normal human hepatocytes). All experiments were performed in triplicate. p-values reported in the graph were calculated by a two tailed t-test.

Selected proteins elevated in patient plasma were tested on three human HCC cell lines by monitoring their effect on the cellular growth rate using the IncuCyte device. Huh7, Hep3B, and HepG2 were incubated with recombinant proteins. Addition of 2 ng/ml and 5 ng/ml FGF2 induced proliferation in all HCC cell lines tested. Moreover, FGF2 at both tested doses induced proliferation of a fourth, confirmatory Hus-E/2 cell line (i.e., immortalized normal human hepatocytes)^18^ (Fig. 2C–F). 1 ng/ml EGF induced proliferation exclusively of Hep3B (Fig. 2B). Addition of 1 ng/ml EGF with 0.1 ng/ml IL-6 induced proliferation of Huh7 cells, but not other lines (Fig. 2A).

### Mouse hepatocytes heat stressed to the conditions of the RF peri-ablational zone secrete pro-tumorigenic factors

**Figure 3:**
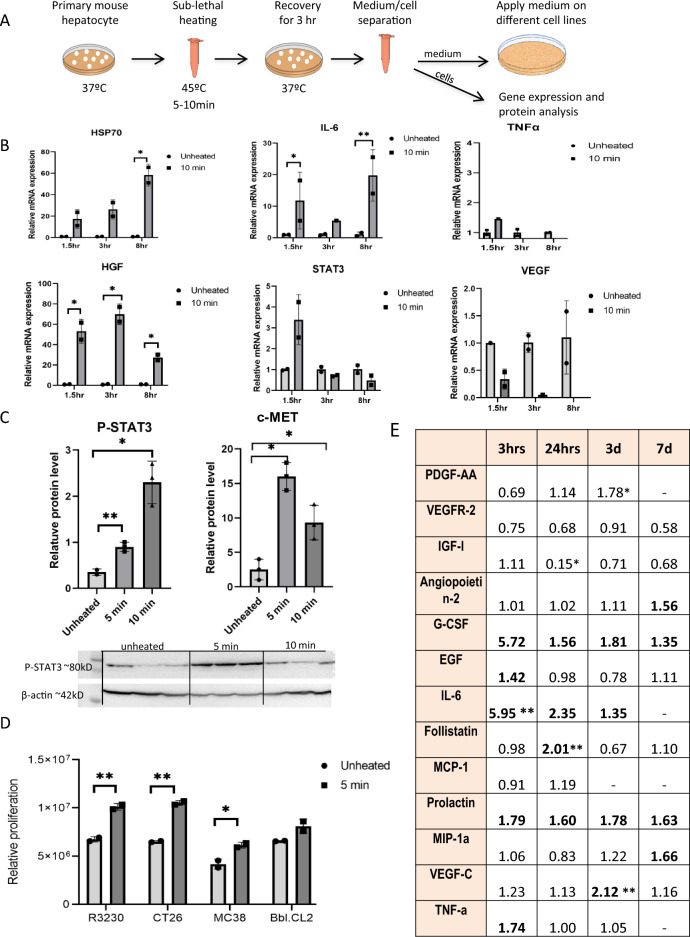
Mouse hepatocytes subject to hyperthermic doses observed in peri-ablational tissue promote tumor cell growth by producing multiple pro-tumorigenic factors. (**A**) Purified primary mouse hepatocytes were subjected to moderate hyperthermia doses 45 °C for 5 or 10 min following 1.5–8 h incubation at 37 °C (n = 12). Medium and cells were collected for further analysis. (**B**) Moderate hyperthermia dose 45 °C × 10 min induces time dependent elevation of HSP70, IL-6, STAT3 and HGF gene expression of mouse primary hepatocytes with maximum expression of each cytokine observed at different incubation times (n = 4, Total = 12). (**C**) Western blot analysis demonstrates increase P-STAT3 and C-met in the heated mouse hepatocytes (n = 3 each arm, Total N = 9). (**D**) Medium derived from primary mouse hepatocytes heated to 45 °C × 5 min followed by incubation for 3 h at 37 °C led to significant increased cellular growth for three unheated cancer cell lines (n = 3, Total N = 18). (**E**) Likewise, multiple growth factors are present in the plasma of mice treated with a single 70 °C × 5 min application of RFA to normal liver, a dose that subjects the peri-ablational rim of hepatocytes to hyperthermic temperature (N = 24). For all experiments p-values calculated by two tailed t-test *p < 0.05, **p < 0.01.

In order to identify pro-tumorigenic activation of the liver parenchyma, we assessed the mRNA expression and protein secretion and activation when subjecting primary hepatocytes isolated from C57Bl/6 mice to moderate hyperthermia in-vitro simulating the in-vivo peri-ablational microenvironment^19–21^. Hepatocytes were heated to 43 °C or 45 °C for 10 min and incubated thereafter at 37 °C for 1.5, 3, and 8h (Fig. 3A). The mRNA expression profile of factors and cytokines implicated in RF-induced tumorigenesis including: IL-6, STAT3, HGF, and VEGF were evaluated by relative quantitative Real-Time PCR (Fig. 3B). Heat shock protein (HSP) 70 was used as a control to detect response to thermal activation^20^, and tumor necrosis factor (TNFα) was used as a control for inflammation^22^. Intracellular transcription factor STAT3, its active phosphorylated form, and c-met were assessed by Western blot. HSP70, IL-6, STAT3, IL-6 receptor and HGF mRNA expression levels were significantly upregulated. TNFα expression showed little, if any, upregulation. VEGF expression showed downregulation (Fig. 3B). Western blot showed an 83 ± 10% increase in phosphorylation of STAT3 at 3 h incubation following 5 and 10 min heating indicating activation of STAT3 (p < 0.05) (Fig. 3C). Western blot also showed upregulation of c-Met protein in heated compared to non-heated cells. These results indicate that RFA activates pathways with pro-proliferative potential well known to play a significant role by heat damaged hepatocytes^8,15,16,23^.

Next, to determine whether moderate hyperthermia induces sufficient secretion of pro-proliferative factors from normal parenchymal cells to induce increased tumor growth, untreated cancer cells were incubated with medium from mouse primary hepatocytes subjected to 45 °C × 5 min and incubated for 3 h at 37 °C. After 48 h incubation at 37 °C, tumor cells were stained with crystal violet to assess the effect on cell proliferation. Medium of heated hepatocytes enhanced growth of several tumor lines in-vitro including R3230 by 30%, CT-26 by 32%, MC38 by 29%, and BNL.CL2 by 22% (Fig. 3D).

C57BL6 mice were then treated with a single 70 °C × 5 min application of RFA to normal liver, a dose that subjects the peri-ablational rim of hepatocytes to hyperthermic temperature. Multiplex ELISA analysis showed high levels of cytokines and growth factors in mice serum post-RFA, indicating early response by secretion of pro-tumorigenic factors 3 h post-RFA including IL-6 (5.95-FC, p < 0.01) and EGF (1.4-FC). A continual response was detected with elevated levels of VEGF 3 days post-RFA (2.2-FC, p < 0.001) and Angiopoietin-2 (1.6-FC) at 7 days (Fig. 3E).

### Thermally stressed murine hepatocytes induce activation of multiple factors including the FGF protein family

**Figure 4:**
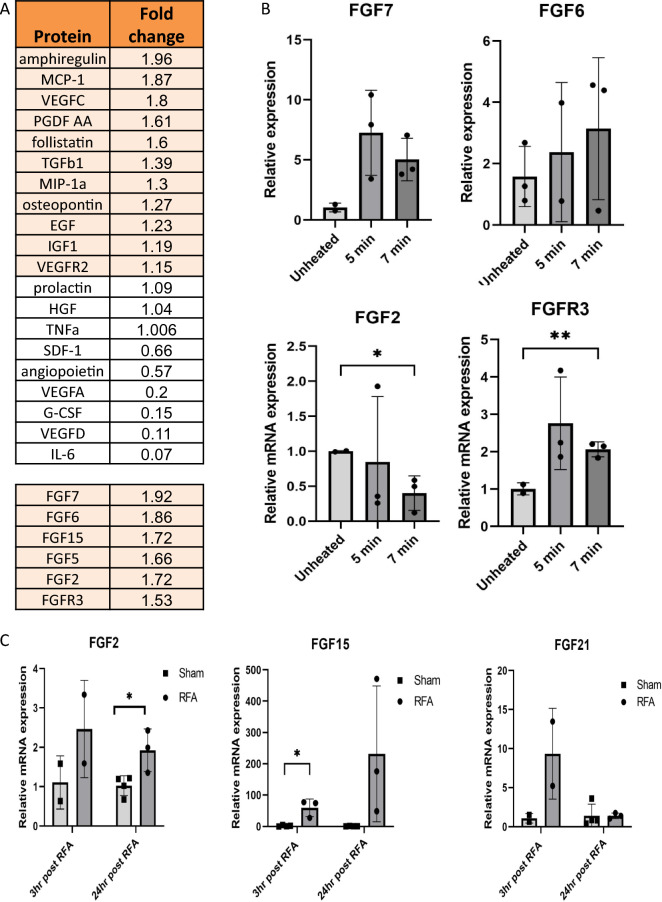
Thermally stressed hepatocytes induce activation of multiple factors including the FGF protein family. (**A**) Proteomics analysis (Somalogic) revealed increased secretion of various factors associated to tumor progression including FGF’s from thermally stressed mouse hepatocytes (n = 6). (**B**) qRT-PCR shows an increase in the mRNA levels of several FGFs 3 h following heating of primary mouse hepatocytes at 45 °C for 5 or 7 min (n = 3 each arm, Total N = 9). (**C**) qRT-PCR on the peri-ablational rim 3 h and 24 h post liver RFA shows high levels of FGF2, FGF21, and FGF15 post ablation at both of the indicated time points (n = 11). For all experiments p-values calculated by two tailed t-test *p < 0.05, **p < 0.01.

Proteomic analysis using the SomaLogic based platform indicated increased secretion of multiple proteins from primary mouse hepatocytes subjected to moderate hyperthermia. Medium of heated hepatocytes to 45 °C for 5 min and recovery of 3 h at 37 °C was analyzed and compared to medium of non-heated hepatocytes that served as a control (Fig. 4A). Most notably, a wide range of proteins from the fibroblast growth factor family including FGF2, FGF7, FGF6, (1.72, 1.92, 1.86 FC, respectively) and other FGFs were increased in the medium of the heated hepatocytes. qRT-PCR showed increased mRNA levels of multiple FGFs 3 h following heating at 45 °C for 5 min or 7 min of primary mouse hepatocytes. FGF7 was significantly increased by 7.6 ± 1.8 fold with 5 min heating and 5.8 ± 09 with 7 min heating. FGFR3 increased significantly at 7 min heating (3.2 ± 1.4). However, FGF2 mRNA levels decreased 3 h post hepatocyte heating (Fig. 4B). qRT-PCR of the peri-ablational rim 3 h and 24 h post a 70 °C × 5 min application of RFA to normal liver, showed elevated levels of FGF2, FGF21, and FGF15 post-ablation at both time points. FGF2 mRNA levels increased by 2.5 ± 0.7-FC 3 h post-RFA and by 2 ± 0.3 (p < 0.01) FC 24 h post-ablation. FGF21 expression increased at 3 h (10.2 ± 4.3), and 24 h post-RFA (2.09 ± 0.1). FGF15 levels also increased 3 h (59.8 ± 16, p < 0.01) and 24 h post-RFA of normal liver (232.1 ± 125) (Fig. 4C).

### Thermally stressed human hepatocytes induce proliferation of HCC cell lines and activation of multiple factors including the FGF protein family

**Figure 5:**
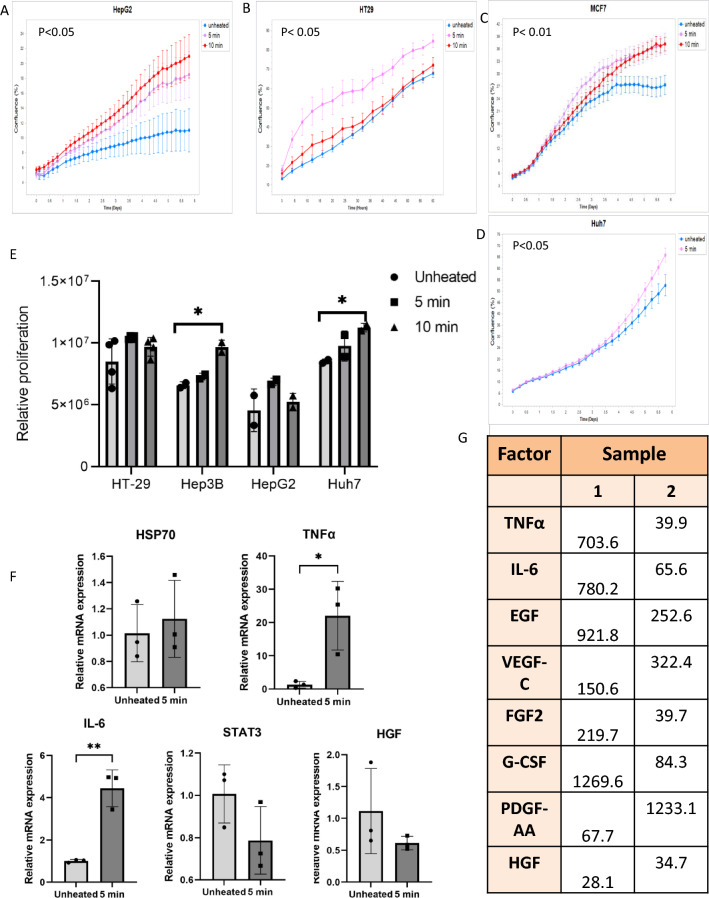
Thermally stressed human hepatocytes induce proliferation of HCC cell lines and activation of multiple factors including the FGF protein family. (**A**–**E**) Medium derived from human immortalized hepatocytes heated to 43 °C × 5 min or 10 min followed by incubation for 3 h at 37 °C led to increased growth when added into the media of several types of unheated human cancer cell lines (N = 10). (**F**) qRT-PCR indicates increase in mRNA levels of TNFα and IL-6 in human immortalized hepatocytes heated to 43 °C × 5 min followed by incubation for 3 h at 37 °C. (**G**) Multiplex ELISA analysis showed high levels of multiple pro-tumorigenic factors secreted by human immortalized hepatocytes subjected to moderated hyperthermia (43 °C × 5 min) and subsequent 3 h 37 °C incubation. For all experiments p-values calculated by two tailed t-test *p < 0.05, **p < 0.01.

To demonstrate applicability to human cells (and confirm results in a second cell line), medium derived from human immortalized hepatocytes was heated to 43 °C × 5 min or 10 min followed by incubation for 3 h at 37 °C. This led to increased growth when added to the media of several unheated human cancer cell lines (Fig. 5A–D). Following the addition of this conditional media, significantly increased cell growth was observed from 24 h to 6 days by the IncuCyte device. HepG2 and MCF7 cells incubated with heated hepatocyte medium significantly increased cell growth at both heating doses (43 °C × 5 min and 10 min). For HT-29 cells, medium of heated hepatocytes enhanced cell growth when adding medium heated for 5 min only (43 °C). Similarly, medium from cells heated 43 °C × 5 min enhanced Huh7 cell growth. Additionally, cell proliferation was observed by crystal violet staining 24 h after incubating the tumor cells lines with conditional medium (Fig. 5E). qRT-PCR on human immortalized hepatocytes heated to 43 °C for 5 min followed by incubation at 37 °C for 3 h indicated significant increases in mRNA expression of factors known to be involved in tumorigenesis post-RFA. TNFα mRNA levels significantly increased by 22.1 ± 5.6 FC 5 min post heating at 43 °C following 3 h incubation at 37 °C (p < 0.05). Similarly moderate hyperthermia induced mRNA expression of IL-6 by 4.9 ± 1 FC (p < 0.01) (Fig. 5F). Milliplex analysis of heated hepatocyte medium (43 °C × 5 min and 3 h incubation at 37 °C) showed increased secretion of all tested proteins including TNFα, IL-6, EGF, VEGF-C, FGF2, G-CSF, PDGF-AA, and HGF (Fig. 5G).

### FGF inhibition attenuated tumorigenesis in-vivo and in-vitro

**Figure 6:**
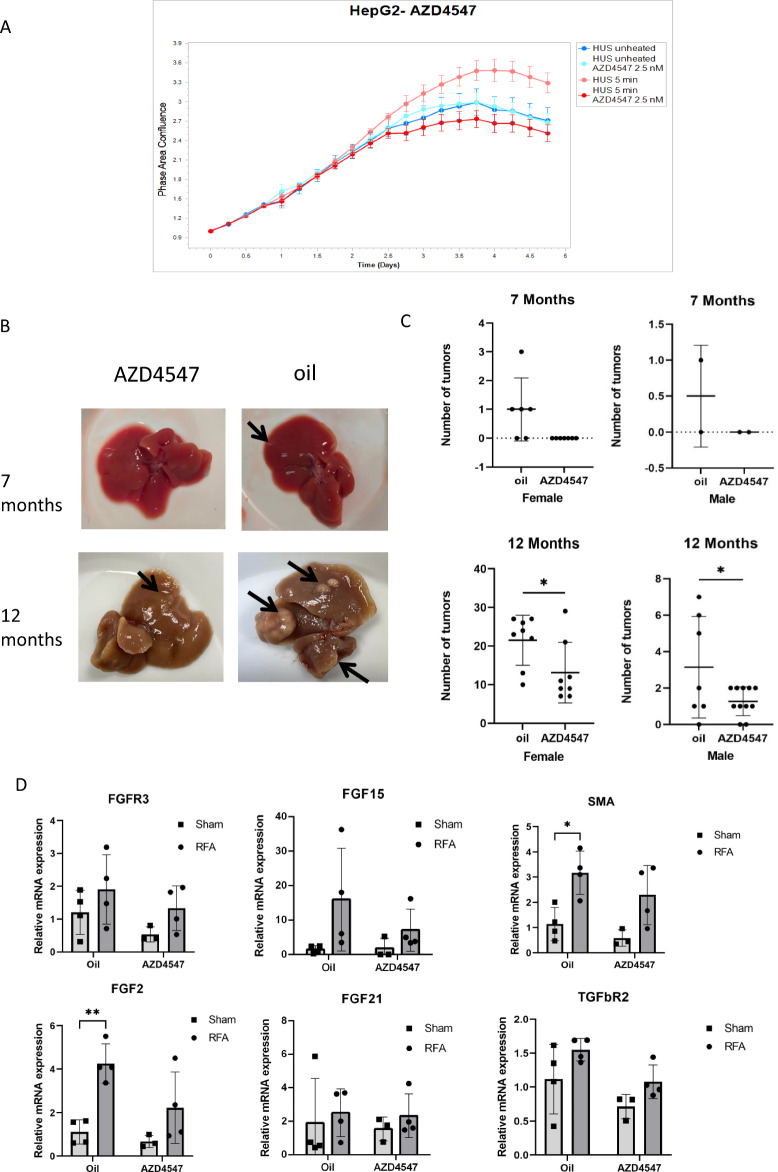
FGF inhibition attenuates tumorigenesis in-vivo and in-vitro. (**A**) The FGFR inhibitor AZD4547 reduced HepG2 cells proliferation induced by medium of heated human immortalized hepatocytes (43 °C for 5 min followed by 3h at 37 °C) (n = 3 each arm, Total N = 12). (**B**) images of the MDR2-KO liver treated with FGFR inhibitor or oil, arrows mark some of the tumors. (**C**) FGFR inhibitor reduced number of liver tumors 1 month post-RFA treatment in 7 months (n = 17) and 12 months MDR2-KO mice (7–11 animals/arm, Total N = 34). (**D**) qRT-PCR on the peri-ablational rim 24 h post liver RFA of MDR2-KO mice with FGFR inhibitor and its control (oil) shows reduced levels of FGFs post inhibitor administration. α-SMA showed increased levels post-RFA in the oil administration that are reduced by FGFR inhibitor (N = 15). For all experiments p-values calculated by two tailed t-test *p < 0.05, **p < 0.01.

In an effort to further assess the role of FGFs on post-RFA tumorigenesis, we initially tested inhibiting FGF signaling in-vitro by using the most clinically translatable FGFR inhibitor agent we could identify. Accordingly, AZD4547, was selected as it is already in clinical oncology trials based upon its activation of FGF signaling pathway^24,25^. HepG2 cells were incubated with the FGFR inhibitor AZD4575 at 37 °C. After 24 h incubation, conditional medium of the Hus-E/2 immortalized human hepatocytes (heated to 43 °C for 5 min minutes following 3 h at 37 °C) was added to HepG2 cell media. The proliferation of HepG2 cells was monitored for 5 days by IncuCyte. Medium of heated hepatocytes induced increased HepG2 proliferation in the absence of the inhibitor, compared to the non heated medium (20%). However, addition of heated hepatocyte medium did not induce increased proliferation of the HepG2 cells incubated in the presence of FGFR inhibitor (Fig. 6A).

In-vivo experiments were performed on MDR2 KO mice (Total N = 51)^10,26^, including 22 males and 29 females that were analyzed separately based upon known tumor growth characteristics. 7 months animals (n = 17) were chosen to investigate the induction of tumor development and 12 months animals (n = 34) to investigate the rate of tumor development. Mice were treated daily with the FGFR inhibitor by gavage 1 day before RFA, the day of RFA, and for 2 days post-RFA. Control animals were given oil (the inhibitor solvent) without the inhibitor by gavage to the same schedule. Liver ablation was performed on all animals. At 30 days post-RFA, liver tumor load was visually assessed (Fig. 6B). A reduction in the number of tumors was observed in animals given the inhibitor compared to the control at both ages. In the 7 months animals, no tumors were observed with the FGFR inhibitor compared to 0.5 ± 0.5 tumors in males and 1 ± 0.5 tumors in females in RFA-treated animals. In 12 months mice, FGFR inhibitor significantly reduced the tumors from 3.14 ± 1.05 to 1.27 ± 0.2 (p < 0.05) in males and from 21.5 ± 2.29 to 13.12 ± 2.76 in females (p < 0.05) (Fig. 6C). qRT-PCR measurement of FGF expression in the liver of MDR2-KO animals 24 h post-RFA that received 2 doses of FGFR inhibitor showed decreases in all tested FGFs both for sham and RFA animals (Fig. 6D). Moreover qRT-PCR measurements in these animals showed that FGFR inhibitor reduced increased expression of α-SMA 24 h post-RFA compared to animals treated with oil alone (p < 0.05) (Fig. 6D).

### Cellular source of FGF proteins in MDR2 KO mice

**Figure 7:**
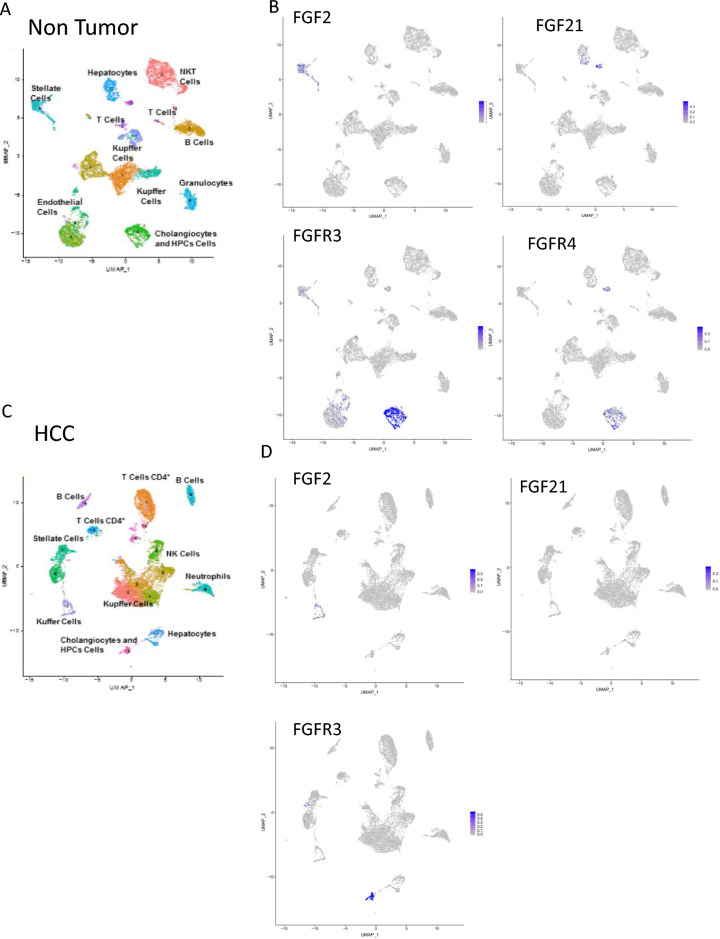
Cellular source of FGF proteins in MDR2 KO mice. (**A**) UMAP presentation showing all cellular clusters in non-tumor liver tissue of 18 month old MDR2-KO mice. (**B**) UMAP presentation showing staining of cells for FGF2, FGF21, FGFR3 and FGFR4 in non-tumor liver tissue of 18 month old MDR2-KO mice. (**C**) UMAP presentation showing all cellular clusters in HCC tumor of 18 month old MDR2-KO mice. (**D**) UMAP representation showing staining of cells for FGF2, FGF21 and FGFR3 in HCC tumor of 18 month old MDR2-KO mice.

In order to investigate the cellular source of members of the FGF protein family, we performed RNA-Seq/single cell analysis (scRNASeq; × 10 analysis) on liver sections of 18 months MDR2-KO mice with a background of inflamed liver and HCC tumors (Fig. 7A,C). This demonstrated that FGF2 in non-tumor tissues is mostly expressed in stellate cells (blue dots in Fig. 7B) and additionally with lower expression in Kupffer cells (Fig. 7B). In tumors, FGF2 expresses in Kupffer cells (Fig. 7D). FGF21 is expressed in hepatocytes both in non-tumor and tumor tissue (Fig. 7B,D). The receptor, FGFR3, has high expression in cholangiocytes and lower expression in endothelial cells, in tumor and non-tumor tissue (Fig. 7B,D). FGFR4 is highest expressed in cholangiocytes with lower expression in hepatocytes in both in non-tumor and tumor tissue (Fig. 7B,D). Moreover, mRNA expression in liver MDR2-KO animals show significant increased levels of a-SMA 24 h post-RFA from 1.14 ± 0.5 to 3.17 ± 0.7 FC (p < 0.05) (Fig. 6D).

## Discussion

In this study, we demonstrate that the FGF family may play a significant role in tumor progression post-RFA, in addition to well-known contributions as inducers of proliferation, angiogenesis, apoptosis escape, and other signaling pathways in multiple cancer types. Moreover, we show that heat-damaged hepatocytes surrounding an ablated area post-RFA may be initiators to pro-oncogenic pathways including via FGF signaling.

Prior studies of pro-tumorigenic effects post-RFA have identified alternate pathways including factors such as c-MET/HGF, IL-6, and STAT3^8,10,11,13,15,16,23,27^. We have previously shown that blocking any of these factors reduces tumor load post-RFA in several rodent models and in-vitro^11^. The cellular response post-RFA may be induced by different populations surrounding the RFA zone such as injured parenchymal cells, or residual tumor cells^8,15,17^. There is also increased migration to the peri-ablational zone, including activated myofibroblasts^14^. Activated myofibroblasts are known to secrete a range of tumorigenic growth factors including HGF^28^. Previous studies confirm that these cells accumulate at the ablated zone as part of the wound healing process in response to unknown signals starting from 72 h post-RFA lasting at least 14 days^14^. We hypothesized that their accumulation to the ablated zone may be triggered by the secreted FGFs from heat-stressed hepatocytes that comprise the dominant parenchymal background. In essence, recruitment of additional cell populations into the ablation zone is responsible for not only reparative processes, but additionally, the cytokines implicated in the RF-induced tumorigenesis cascade^28^. Thus, elucidating their precise role in the initiation of multiple pathways that may induce the ‘off-target’ effects of RFA may offer not only greater mechanistic insight, but also better overall targets for therapeutic strategies.

Plasma analysis of HCC and CRC patients revealed a wide range of proteins, specifically growth factors, 90 min post-RFA treatment. 70–80% of the liver is comprised of parenchymal tissue, mostly hepatocytes^29^. Ablation of a rim of these is necessary for successful treatment with the implication that heat-stress of normal parenchymal cells will be an integral by-product of any successful thermal ablation. As we observed an immediate response post liver RFA prior to cellular recruitment, we hypothesized that initiation of tumorigenesis is mediated mainly by the hepatocytes. Accordingly, we aimed to elucidate a more complete picture of the responses originating from liver parenchymal that may induce tumorigenesis post-RFA. To accomplish this, an in-vitro, unbiased approached was applied. A protein microarray on medium of heated mouse hepatocytes and confirmatory human hepatocytes subjected to moderate hyperthermia indicated a significant increased secretion of growth factors, specifically fibroblast growth factors.

Plasma of patients treated by RFA displayed a large variety of expression of multiple growth factors. We attribute this heterogeneity of response to the fact that several cell populations are likely to be affected by moderate hyperthermia as part of liver ablation. Moreover, in addition to the cell types that might be activated, both the volume of effected cells and amount of heat stress they see is variable based upon clinical application of RFA which can potentially also change the profile of the activated factors in each patient. Additionally, this observed variety may also indicate that the proteins we detected in the serum are not all the factors that are produced post-RFA, and that some factors may be consumed locally and/or have a direct local effect. However, as shown, medium of mouse and human heated hepatocytes and plasma of the patients post-RFA selectively induced selective proliferation of different types of HCC cell lines. This indicates both that several pathways and different factors can promote tumor proliferation in response to heat damage^9,16,21,30^ and that simultaneously that different lines of cancer cells are sensitive to different growth factors and activate proliferation or other pathways^31^. Some of the detected factors have been previously identified as tumorigenesis mediators post-RFA. Yet, here we additionally identify the FGF protein family, which may also play a predominant role.

It has been shown previously by different groups that serum levels of FGF2 are elevated in cirrhosis and HCC patients^32^. It is also known that high levels of FGF19/FGFR4 signaling pathway have been associated with poor outcomes in patients with HCC^33–35^. In our studies, we demonstrate increased levels of FGF2 in plasma of patients post-RFA in addition to elevated levels of several FGFs in mice liver post-RFA including FGF19 mouse ortholog FGF15. It is well known that the FGF signaling pathway through MAPK cascades plays an important role in survival of HCC cell lines^36^. Our results demonstrate that the FGF signaling pathway is activated post-RFA from heat damaged hepatocytes, and we confirm this by showing secretion of FGF2 in plasma of some patients 90 min post-RFA. This indicates an immediate response that may initiate tumorigenesis post-RFA by activating the MAPK signaling cascade, by increasing p-ERK, a pathway shown to be active post-RFA in colorectal and other cancers^36–39^. Moreover, FGFs play a pivotal role in liver fibrosis by activating hepatic stellate cells^40^. In humans, FGF2 may be expressed in hepatocytes and stellate cells in additional to other cells^32,41,42^. Here, we demonstrate that in the MDR2-KO mouse model of chronic liver inflammation that culminates into hepatocarcinogenesis that FGF21 is expressed in hepatocytes and FGF2 is specifically expressed in stellate cells which may indicate its role in liver fibrosis in chronic liver inflammation. Thus, proteins of the FGF family may be expressed by several types of liver cells, especially by stellate cells and hepatocytes. However, the FGFRs are mainly expressed in cholangiocytes. Although this requires further investigation, we suggest that RFA induces FGF’s activation which leads to liver fibrosis as indicated by an increase in mRNA levels of α-SMA. We further show that the FGFR inhibitor AZD4547 reduced the increased heated hepatocyte medium induced proliferation of HepG2 cells by blocking FGFR2. Our group has previously demonstrated that RFA induced early tumor development and increased the number of tumors in Mdr2-KO mice, a model with acute liver inflammation that eventually develops liver tumors^10^. Here, we also show that the FGFR inhibitor AZD4547 given as a daily dose for 2 days before and after RFA reduced the number of liver tumors 30 days post ablation in both 7 and 12 months mice. These results indicate that blocking the FGF signaling pathway coincidentally with the RFA treatment, meaning inhibiting a potential key mediator at the initiation of the signaling cascade post ablation, can facilitate the treatment to reduce the tumorigenesis post-RFA. These results have major clinical implications, demonstrating that adding an FGFR inhibitor can significantly reduce the pro-oncogenic effect observed in some cases post-ablation. Eliminating this unwanted effect by adding an FGFR inhibitor or other blockers to RFA treatment in a select cohort of patients might be beneficial or even necessary to improve the clinical outcomes.

There are some limitations to our research regarding our understanding of induced pro-tumorigenic pathways post-RFA. First, additional factors such as PDGF-AA and G-CSF were upregulated post ablation in the protein analysis performed, and these will likely require further investigation as to their precise role in the pro-oncogenic post-RFA cascade. We are aware of the possibility that additional cells may secrete critical mediators and therefore aim to continue investigating the role of other cellular populations recruited to the peri-ablational zone after RFA treatment. Yet, our results reinforce the need to potentially elucidate additional pathways and key mediators, as our results strongly suggest that many pathways may be activated depending on the tumor type and the amounts and types of normal parenchymal cells heat stress which varies clinically from patient to patient. Moreover, it has been shown that the coagulative necrosis of tumor tissue and the production of local tumor protein debris may contain a large number of various antigens, which can stimulate a specific cellular immune response^25^. Thus, study of the immunologic effects of tumor ablation, their underlying mechanisms, and how this too may alter clinical outcomes is likely warranted. Moreover, other ablation methods including thermal and chemical may also induce unknown pro-tumorigenic pathways. Thus, identifying an additional targetable pathway may offer additional options for therapy tailored to appropriate patient populations. This may lead to the need for better patient profiling with biomarkers before or after ablation, an additional area likely worthy of further study.

In conclusion, we demonstrate a primary signaling pathway induced by RFA that plays a substantial role in the initiation of the tumor recurrence and propagation. We show that FGFs are secreted in to the plasma of HCC and CRC patients 90 min post-ablation. Moreover, FGFs are secreted from heat damaged hepatocytes in-vitro. Additionally, we demonstrate that plasma and medium containing FGFs induce proliferation of tumor cell lines. Finally, administering FGFR inhibitor reduces tumor proliferation post-RFA. As we suggest, FGFs may be initiators to a cascade of several signaling pathways including, RAS-MAPK-ERK, PI3K-AKT, TGFβ, WNT signaling and others that are known to regulate epithelial-to-mesenchymal transition (EMT) and invasion^30,36,43–46^. Given that many parallel pathways can induce proliferation and tumor progression post-RFA, identifying the earliest initiators of this cascade such as hepatocyte (or other parenchymal cell) produced FGF and blocking it at the earliest point may be crucial to successfully eliminating these tumorigenesis effects.

## Materials and methods

### Overview

First, the proliferative effect of plasma of 3 HCC and 6 CRC patients 90 min post liver RFA was investigated on human HCC cell lines HepG2, Huh7 and Hep3B. A multiplex ELISA protein analysis identified potential plasma cytokines and growth factors upregulated by RFA. To confirm specific factors proliferative effects, recombinant proteins of the upregulated proteins observed post-RFA were incubated with human HCC cell lines, as measured by the IncuCyte device (Sartorius, USA). Next, primary mouse hepatocytes and immortalized human hepatocytes^18^ were subjected to moderate hyperthermia (43–45 °C) to mimic the thermal stress induced during ablation in normal parenchymal cells^11^. Medium of both human and mouse heated hepatocytes (conditional medium) was added to the incubation medium of multiple tumor cell lines including HCC, CRC, and breast adenocarcinoma and tumor cell proliferation was monitored^11^. The heated hepatocytes medium was then analyzed by protein array and multiplex ELISA to detect factors secreted post heating. Finally, given increased secretion of fibroblast growth factors including FGF2, FGF7, and FGFR3 post-heating in-vitro and in plasma of human and mice post-RFA treatment, we used an FGFR inhibitor to block the proliferation observed post-RFA in-vivo and post heating in-vitro**.**

### Patient population

This institutional review board-approved, prospective study consisted of 9 consecutive patients diagnosed with either primary HCC (n = 3) or liver metastases of colorectal cancer (n = 6) (see Table S1 for additional demographics and 3 and 12 month clinical outcome). All patients were treated with multi-tined RF ablation (RITA, Angiodynamics, Marlborough, MA) according to manufacturer protocol at the Department of Radiology and Nuclear Medicine, University Hospital Brno and Masaryk University, Brno, Czech Republic. Plasma was collected pre RFA and 90 min post RFA. All the patients provided signed, informed consent approved by the institutional ethical committee of the University Hospital of Brno (in 2018). All methods were performed in accordance with the relevant guidelines and regulations. All biological specimens were anonymized prior to laboratory analysis^47^.

### Cell culture

R3230 (rat mammary carcinoma) (Center for Molecular Imaging, Harvard medical school, MA, USA), CT-26 (mouse CRC) (ATCC, Virginia, USA), MC38 (mouse CRC)^48,49^ and HT29 (human CRC) (ATCC, Virginia, USA) cells were grown at 37 °C in 5% CO_2_ in RPMI medium supplemented with 10% fetal calf serum (FCS) (Biological Industries, Israel), 100 IU/ml penicillin, 100 mg/ml streptomycin, and 2% l-glutamine (Thermo Fisher Scientific, MA, USA). Bnl.CL2 (mouse HCC) (ATCC, Virginia, USA), Hepa1.6 (mouse HCC) (ATCC, Virginia, USA) and Huh7 (human HCC) (NIBIOHN, JCRB Cell Bank, Japan) cell lines were grown in DMEM medium supplemented with 10% FCS (Biological Industries, Israel), 100 IU/ml penicillin, 100 mg/ml streptomycin, and 2% l-glutamine (Thermo Fisher Scientific, MA, USA). MCF7 (human mammary carcinoma) (ATCC, Virginia, USA), HepG2 and Hep3B (both HCC) (ATCC, Virginia, USA) cell lines were grown in EagleMEM medium supplemented with 10% FCS (Biological Industries, Israel), 100 IU/ml penicillin, 100mg/ml streptomycin, and 2% l-glutamine (Thermo Fisher Scientific, MA, USA). All cell lines tested negative for Mycoplasma. Primary mouse hepatocytes were grown in DMEM/F-12 medium supplemented with 100 units of ITS (I1884, Sigma-Aldrich, Rehovot, Israel) of 10% fetal calf serum (FCS) (Biological Industries, Israel), 100 IU/ml penicillin, 100 mg/ml streptomycin, and 2% l-glutamine (Thermo Fisher Scientific, MA, USA). Human immortalized hepatocytes cell line (Hus-E/2) we received kindly from Prof. Kunitada Shimotohno’s lab (Kyoto University, Japan) the cells were grown according the recommended protocol^18^.

### Hepatocytes isolation by liver perfusion

Primary mouse hepatocytes were isolated from 8- week-old C57BL6 male mice by liver perfusion^50^ using LiberaseTM research Grade (SigmaAldrich, Rehovot, Israel), and purified by Percoll gradient (Santa Cruz biotechnologies, Texas, USA).

### In-vitro moderate hyperthermia heating assays

5ml medium was pre-heated in a water bath for ~ 20 min to achieve a regulated temperature of 45 ± 1 °C or 43 ± 1 °C. A 50 ml conical tube containing 1 × 106 cells in 50 µl was incubated in the water bath for 5–10 min. 200 µl of pre-heated medium was added in order to rapidly bring the cells to the desired temperature. At the conclusion of heating, 50 µl room temperature standard culture medium was added. Cells were subsequently incubated at 37 °C from 1.5 to 24h. Control cell suspensions were incubated continuously at 37 °C.

### mRNA expression analysis

For quantitative real-time PCR, RNA was purified from heated cells using standardized Trizol RNA extraction protocols. Expression of IL-6, TNFα, STAT3, HGF, VEGF and all FGFs was tested using the CFX384 TouchTM Real-Time PCR Detection System (BioRad, California, USA). Additionally, HPRT, GAPDH (ubiquitous endogenous housekeeping genes) and HSP70 expression were assessed to document normal cellular functioning and response to heating, respectively. Results of mRNA expression are expressed as ratio to the mRNA present in unheated cells and HPRT or GAPDH controls.

### Quantification of proteins

The levels of proteins in the plasma of HCC and CRC patients were determined using a Human magnetic premixed multi-analyte luminex assay kit (R&D Systems, Minneapolis, USA). Plasma of the patients was collected pre-RFA, 90 min and 24 h post RFA. The same assay was used to analyze the levels of proteins secreted by human immortalized hepatocytes subjected to moderate hyperthermia (43 °C × 5/10 min following 3 h at 37 °C). The levels of proteins in mice plasma post RFA were determined by the MILLIPLEX MAG mouse angiogenesis/growth factor magnetic bead array panel (#MAGPMAG-24K, EMD Millipore Corporation, MA, USA) combined with Magnetic Luminex Assay (LXSAMSM, R&D Systems, Minneapolis, USA) detection method as per manufacturer's protocol. Proteins tested included: PDGF-AA, VEGF-R, IGF-I, Angiopoietin-2, G-CSF, EGF, IL-6, Follistatin, MCP-1, Prolactin, Mip-1α, VEGF-C and TNFα. For in-vivo studies, C57BL/6 mice were treated with RFA or sham procedures. Animals were sacrificed 3 h, 24 h, 3 days and 7 days post treatment. Blood samples were collected and centrifuged (3000*g*; 10 min; 25 °C) (MegaFugeTM 16R, ThermoFisher, MA, USA) to purify the plasma. The detection of the proteins secreted by heated mouse hepatocytes (45 °C × 5 min following 3 h at 37 °C) were determined by the SomaScan platform (Somalogic Inc. Denver, Colorado, USA). c-Met and STAT3 quantification were performed using Western blot analysis. Heated cells were homogenized using RIPA buffer (Abcam, Cambridge, UK). Protein was quantified by Bradford protein assay with 40mg total protein loaded on 10% SDS–polyacrylamide gels and blotted onto PVDF transfer membranes. Nonspecific binding was blocked with 1% skim milk for 1 h followed by incubation with c-Met 1:100 (SC-162; Santa Cruz Biotechnology, Texas, USA), P-STAT3 1:100 (#9131-Cell signaling tech, Danvers, USA) and STAT3 1:1000 (SC-8019; Santa Cruz Biotechnology) antibodies. Standardization of protein quantities was performed using β-actin with results for the assessed protein expressed as the ratio to control specimens. All samples and standards were measured in triplicates.

### In-vitro tumor cells growth assessment

To prepare conditional medium (i.e. media containing factors excreted by tumor cells subject to moderate hyperthermia), human hepatocyte cell lines were heated at 43 ± 1 °C and mouse primary hepatocytes at 45 ± 1 °C for either 5 min or 10 min and subsequently incubated at 37 °C for 3 h. Controls were prepared in a similar manner, with incubation at 37 °C throughout. The effect of conditional medium on cell growth was assessed by an IncuCyte detection system (Sartorius, USA) for 1.5–6 days. Untreated cells were incubated in growth medium without FCS and conditional media was added v/v (final 2.5% FCS). All experiments were performed in triplicate. Cell growth was additionally assessed by crystal violet assay. Cells were incubated with 50% conditional medium diluted in standard culture medium. After 24–48h, cells were washed and stained by crystal violet (Sigma-Aldrich, Rehovot, Israel). Number of cells was assessed by surface covering determined by a plate reader. For further proliferation assays, recombinant proteins were added to the standard culture medium of the cells. The concentration of recombinant protein added was 1 ng/ml for EGF, (AF-100-15, PeproTech, Rocky Hill, NJ), 0.1 ng/ml for IL-6 (AF-200-06, PeproTech, NJ), and 2–5 ng/ml for FGF2 (AF-100-18B, PeproTech, NJ). Cellular growth rate was determined by the IncuCyte device.

### Animal models

Mice were maintained under specific pathogen-free (SPF) conditions in an animal facility with a temperature of approximately 23 °C in a 12-h light–dark cycle and received sterile commercial rodent chow and water ad libitum. Maintenance of mice and all experimental procedures were performed by the Institutional Animal Care and Use Committee approved animal treatment protocols (license number OPRR-A01-5011). All animal experiments were performed after stringent ethical consideration and approval with experiments regulated by veterinarian specialists of the National council for experiment animal experimentation from the Hebrew university of Jerusalem. All experiments were performed, in accordance with ARRIVE guidelines, by individuals with experience in performing RF thermal ablation, and surgery in this model. For all procedures, anesthesia was induced with intraperitoneal injection of a ketamine (50 mg/kg) and xylazine (5 mg/kg). RF ablation was performed in the middle portion of the left liver lobe of WT 8 weeks old male C57BL6 mice. For the HCC animal model, MDR2 KO mice, RF was applied on 7 months (n = 17) and 12 months (n = 34) animals, by placing a 21-gauge needle with a 1-cm exposed tip on the liver, with energy titrated to ensure a mean tip temperature (± standard deviation) of 70 °C ± 1 (approximately 1 W/4 mA) for 5 min using a clinically available RF generator (CC-1 Cosman coagulator system; Radionics, Burlington, Mass). Sample sizes for our primary FGFR inhibitor studies on 12 months MDR KO (i.e. the time at which tumors are only seen in the presence of RF induced tumorigenesis^10^) were based upon Power calculations that determined that a minimum of 7 animals in each of the 4 groups (RF + drug, RF alone for males and females) had an 80% chance of detecting a clinically relevant difference in tumor number at a p < 0.05.

### FGFR inhibitor

For in vivo experiments, mice were given FGFR inhibitor AZD4547 (Abcam, Cambridge, UK) 12.5 mg/kg diluted in 200 μl sesame oil by gavage, 1 dose the day before RFA, 1 dose the day of the ablation, and a third and fourth dose the two next days after RFA^51^. For inhibition of FGFR signaling by AZD4547 in vitro, HepG2 cells were incubated with 2.5 nM AZD4547 concentration. 24 h later, medium of heated human hepatocytes (43 °C × 5 min following 3 h at 37 °C) was added to the cells. Cellular proliferation was monitored by IncuCyte device for 5 days.

### Single cell RNA-Seq (scRNA-Seq) data processing and integration

Raw reads of each sample were processed using the 'count' command of the Cell Ranger software, v2.0.2, aligning the reads to the mouse 10 mm (GRCm38) genome version. The generated report was used for assessing the quality of the samples in terms of cell numbers (14,295, and 12,977), the average reads per cell (20,810 and 13,749), the fraction of reads in cells (94.3% and 87.4%), alignment rate and saturation (51.6% and 31.9%) for HCC and Non-tumor samples, respectively. The datasets were further filtered to retain only high-quality cells with > 200 expressed genes, and < 5% for HCC and 10% for non-tumor of mitochondrial RNA transcripts. Genes expressed in less than 3 cells were excluded. Finally, 11,485 HCC and 11,164 Non-tumor cells were retained for analysis using the package Seurat 3.0.2 and Seurat 4.0.4^52^ on R3.6.3 and R4.0, respectively. Datasets were normalized using “LogNormalize”, a global-scaling normalization method. From each sample, 2000 highly variable genes were selected for the downstream analysis. The 3 datasets were then subjected to scaling, centering, dimension reduction, generating nearest neighbor graphs using PCA1 to PCA20, clustering at 0.6 and visualization by Uniform Manifold Approximation and Projection (UMAP) for both of the samples. Figures to visualize the clusters, as well as the marker expression in the low-dimensional space (UMAP) and the violin plots, were generated by Seurat visualization functions. Cluster cell type identification was done using published markers^53^. Some immune clusters were identified by comparisons within the Immunological Genome Project (ImmGen; https://www.immgen.org/^54^.

### Statistical analysis

For in-vitro moderate hyperthermia experiments, each individual group result was averaged and standard deviation calculated using GraphPad Prism 6.02 software (GraphPad Software, Inc). For IncuCyte experiments, the results are presented as graph as measured and calculated by IncuCyte software (Sartorius, USA). For in-vivo protein arrays data was shown as fold change (FC) compared protein levels post sham operations. All p-values were calculated by a two tail t-test. For animal studies, the metric of tumor number was used as this parameter is a primary determinant as to which interventional oncologic therapy (i.e. ablation or embolization) is appropriate.

### Supplementary Information

Supplementary Information.

## Data Availability

All data generated or analyzed during this study are included in this manuscript.
