# Supplementary material for: Fibroblast growth factors induce hepatic tumorigenesis post radiofrequency ablation
Source: Sci Rep. 2023 Sep 28;13:16341. doi: 10.1038/s41598-023-42819-2 (PMC10539492; doi:10.1038/s41598-023-42819-2)
Supplement: Supplementary file 1 — Supplementary Information. [file 41598_2023_42819_MOESM1_ESM.docx]

**Supplementary material**

**Fibroblast growth factors induce hepatic tumorigenesis post radiofrequency ablation**

Aurelia Markezana^1^, Mor Paldor^1^, Haixing Liao^1^, Muneeb Ahmed^2^, Elina Zorde-Khvalevsky^1^, Nir Rozenblum^1^, Matthias Stechele^1,5^, Lukas Salvermoser^1,5^, Flinn Laville^1,5^, Salome Goldmann^1^, Nofar Rosenberg^1^, Tomas Andrasina^4^, Jens Ricke^5^, Eithan Galun^1^, and Shraga Nahum Goldberg^1,2,3^

**^1^**The Goldyne Savad Institute of Gene and Cell Therapy, Hadassah Hebrew University Hospital, Ein Karem, Jerusalem, Israel;

**^2^**Laboratory for Minimally Invasive Tumor Therapies, Department of Radiology, Beth Israel Deaconess Medical Center (BIDMC), Harvard Medical School, Boston, MA, USA;

^3^ Division of Image-guided Therapy and Interventional Oncology, Department of Radiology, Hadassah Hebrew University Hospital, Jerusalem, Israel.

^4^ Department of Radiology and Nuclear Medicine, University Hospital Brno and Masaryk University Brno, Brno, Czech Republic.

^5^ Department of Radiology, University Hospital, LMU Munich, Germany.

**Supplementary Figure 1: Full-length images of the cropped blots presented in the main figures.**


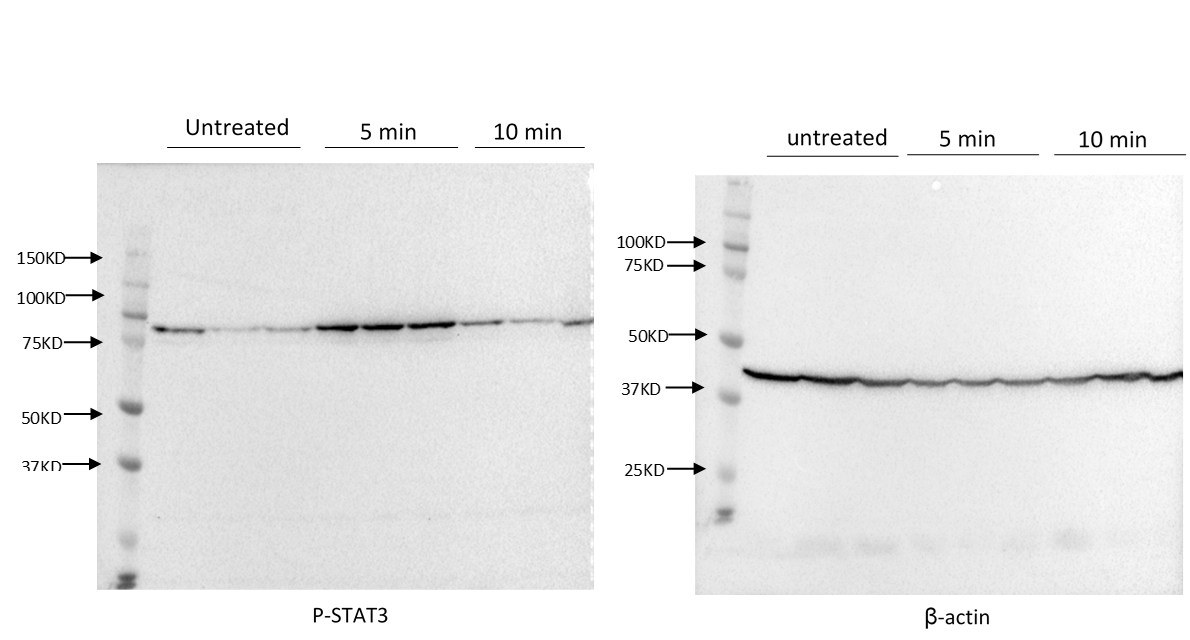
Full-length images of Figure 3C.

**Supplementary Table 1:** **Patient characteristics of those undergoing clinically indicated hepatic RFA.**

mCRC = intrahepatic metastatic colorectal cancer, HCC = hepatocellular carcinoma. CR= complete response, PD = progressive distant intrahepatic disease, PR = viable tumor in ablation margin. TACE=transarterial chemoembolization.

| **Patient** | **Diagnosis** | **SEX**  **(M/F)** | **Age**  **(years)** | **Number of Tumors Ablated** | **Maximum Tumor Diameter (cm)** | **Total Tumor Volume (ml)** | **3 month Follow-up** | **12 month Follow up** |
| --- | --- | --- | --- | --- | --- | --- | --- | --- |
| 1 | HCC | F | 78,5 | 1 | 2,4 | 7 | CR | CR |
| 2 | mCRC | M | 71,3 | 1 | 2,7 | 19 | CR | PD |
| 3 | mCRC | M | 70,3 | 1 | 2,2 | 5,3 | PD (3 new metastases) | Stable from 3m follow-up |
| 4 | mCRC | M | 39,3 | 1 | 1,55 | 1,1 | PR (multiple mets left lobe treated surgically at 4m) | Patient died 9 month post RFA |
| 5 | mCRC | M | 64,7 | 1 | 2,7 | 4,9 | PR | CR (after 2 additional RFA treatments) |
| 6 | HCC | F | 76,3 | 1 | 1,2 | 1,4 | CR | CR (after 2 sessions of TACE) |
| 7 | HCC | M | 68,2 | 1 | 2,7 | 8 | CR | CR |
| 8 | mCRC | M | 76,5 | 1 | 1,5 | 0,7 | PD (6 new metastases) | PD |
| 9 | mCRC | M | 49,1 | 3 | 4 | 12,2 | PD (2 new mets) | PD (after 2 ablations, viable margins) |
